# INFLECT: an R-package for cytometry cluster evaluation using marker modality

**DOI:** 10.1186/s12859-022-05018-w

**Published:** 2022-11-16

**Authors:** Jan Verhoeff, Sanne Abeln, Juan J. Garcia-Vallejo

**Affiliations:** 1 Department of Molecular Cell Biology & Immunology, Amsterdam Infection & Immunity Institute and Cancer Center Amsterdam, Amsterdam UMC, Vrije Universiteit Amsterdam, De Boelelaan 1108, 1081 HZ Amsterdam, The Netherlands; 2 Department of Computer Sciences, Center for Integrative Bioinformatics (IBIVU), Vrije Universiteit Amsterdam, De Boelelaan 1105, 1081 HV Amsterdam, the Netherlands

**Keywords:** Mass cytometry, Data analysis, Clustering results, Clustering evaluation, Unimodality, Software, Phenotyping

## Abstract

**Background:**

Current methods of high-dimensional unsupervised clustering of mass cytometry data lack means to monitor and evaluate clustering results. Whether unsupervised clustering is correct is typically evaluated by agreement with dimensionality reduction techniques or based on benchmarking with manually classified cells. The ambiguity and lack of reproducibility of sequential gating has been replaced with ambiguity in interpretation of clustering results. On the other hand, spurious overclustering of data leads to loss of statistical power. We have developed INFLECT, an R-package designed to give insight in clustering results and provide an optimal number of clusters. In our approach, a mass cytometry dataset is overclustered intentionally to ensure the smallest phenotypically different subsets are captured using FlowSOM. A range of metacluster number endpoints are generated and evaluated using marker interquartile range and distribution unimodality checks. The fraction of marker distributions that pass these checks is taken as a measure of clustering success. The fraction of unimodal distributions within metaclusters is plotted against the number of generated metaclusters and reaches a plateau of diminishing returns. The inflection point at which this occurs gives an optimal point of capturing cellular heterogeneity versus statistical power.

**Results:**

We applied INFLECT to four publically available mass cytometry datasets of different size and number of markers. The unimodality score consistently reached a plateau, with an inflection point dependent on dataset size and number of dimensions. We tested both ConsenusClusterPlus metaclustering and hierarchical clustering. While hierarchical clustering is less computationally expensive and thus faster, it achieved similar results to ConsensusClusterPlus. The four datasets consisted of labeled data and we compared INFLECT metaclustering to published results. INFLECT identified a higher optimal number of metaclusters for all datasets. We illustrated the underlying heterogeneity within labels, showing that these labels encompass distinct types of cells.

**Conclusion:**

INFLECT addresses a knowledge gap in high-dimensional cytometry analysis, namely assessing clustering results. This is done through monitoring marker distributions for interquartile range and unimodality across a range of metacluster numbers. The inflection point is the optimal trade-off between cellular heterogeneity and statistical power, applied in this work for FlowSOM clustering on mass cytometry datasets.

**Supplementary Information:**

The online version contains supplementary material available at 10.1186/s12859-022-05018-w.

## Background

It has been widely accepted in (mass) cytometry that high-dimensional datasets are best approached through unsupervised clustering algorithms [1, 2], revealing structure in high-dimensional space that is not well identified through bivariate sequential gating. Prior to the introduction of unsupervised clustering into flow cytometry, the gold standard in cell classification of conventional flow cytometry data involved manual gating. In this approach, researchers use manually defined coordinates (gates) to group cells in bivariate plots. This process is repeated in a sequential manner until all desired populations have been defined. Manual gating requires a priori knowledge, it is not easily scalable, it is susceptible to the observer’s experience and personal bias, and it misses on untargeted populations. Therefore, there has been a keen interest in the cytometry community to develop automated data analysis methods, such as unsupervised clustering. Opposite to manual gating, unsupervised clustering algorithms do not prioritize any of the given input parameters. Interpretation and visualization of the clustering results is often aided by dimensionality reduction. Additionally, these data-driven algorithms provide more reproducible results, removing researcher bias that comes with manually setting gates in bivariate plots. Since the publication of the first clustering method for cytometry data in 2007, many clustering algorithms have been published and their performance thoroughly compared [3–7]. However, every high-dimensional analysis method makes assumptions on the underlying data that need to be understood by the researcher implementing these methods [8, 9]. The added complexity of clustering algorithms has led to shifting of ambiguity from gating to ambiguity surrounding clustering results. The question of how many meaningful clusters exist in a high-dimensional dataset has proven to be very difficult to answer. Methods such as FlowGrid [7] or Phenograph [10] have automated cluster detection relying on intra-cluster (dis)similarity. However, subsets of rare cells often have high clinical relevance with little phenotypic distance to other subsets. Clustering algorithms specifically designed to capture rare subsets [11, 12] or relying on over-clustering [9] present other limitations: a large number of (small) clusters leads to a loss of statistical power due to corrections for multiple testing. Automated metaclustering, the grouping of phenotypically similar clusters, is a solution to over-clustering, but it is in turn susceptible to the same challenges as one-step clustering methods. ConcensusClusterPlus, the default metaclustering method implemented in FlowSOM, often has a very conservative result, leading to broad clusters encompassing multiple cell types.

Manual identification and optional supervised metaclustering is a laborious process but currently unavoidable when analyzing over-clustered high-dimensional data. It has become common for mass cytometry data to be presented in a way that classifies multiple found clusters as a single phenotype. Clusters identified in an unsupervised manner are grouped together, implying that these (sub-)clusters represent different states of the given phenotype [13–17]. If combined into a larger single cluster, few methods exist to evaluate the results that come from any given clustering algorithm [18, 19], meaning researchers visually inspect heatmaps of median cluster expressions or assess concordance of unsupervised clustering results to dimensionality reduction techniques such as t-SNE or UMAP. Furthermore, none of these approaches address the question of how to set the limit to the number of clusters (or metaclusters) that is adequate for each dataset.

Here, we aim to address this question by a computational method, INFLECT, that iteratively evaluates metaclustering performance to find the highest level of parameter unimodality, and lowest expression spread per metacluster. Evaluating clustering results using these 2 characteristics is based on the assumptions that a multi-modal univariate distribution contains multiple, possibly overlapping, cell populations. A wide marker spread similarly indicates poor clustering. These multiple cell populations can be related, but represent different activation states of a large cell phenotype, or transitional cells differentiating. In turn this means that if a clustering process results in narrow unimodal marker distributions across all generated clusters, it has successfully captured the full cellular heterogeneity in the dataset. INFLECT uses FlowSOM [20, 21] for upstream clustering because of fast runtimes and widespread use. In concordance with the FlowSOM workflow, datasets are overclustered in initial SOM-clustering, and the subsequent metaclustering steps are investigated. Results of the metaclustering are evaluated based on the marker expression distributions within formed metaclusters. For each marker, INFLECT tests for unimodality and assesses marker expression spread. Unimodality is determined through the dip test [22], a statistical test. Marker distribution spread is evaluated through the interquartile range and fails if this range is too high.

## Implementation

**Fig. 1:**
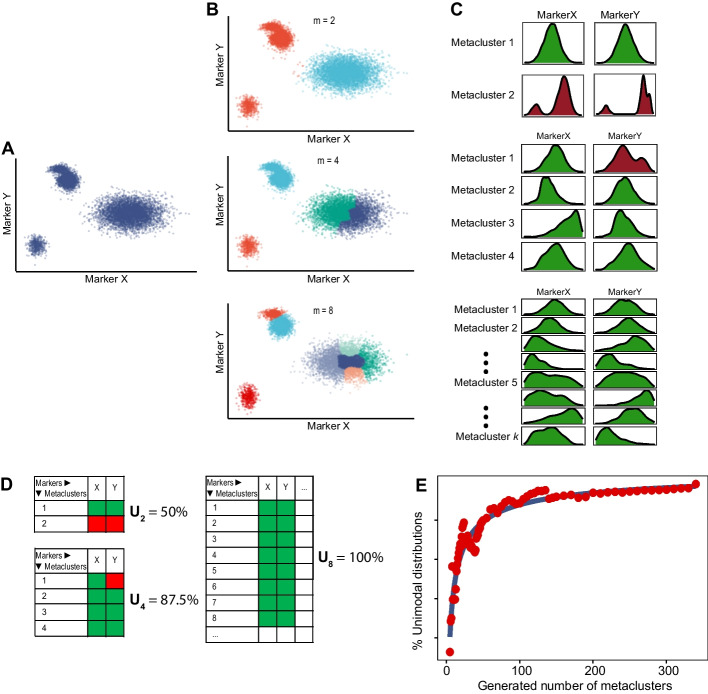
Illustrated representation of the algorithms workflow. **A** A simple 2-dimensional example, based on a dataset containing two markers with 4 populations, 2 of which are connected, is clustered in 2 < *m* < *k* populations. Each clustering result is inspected by assessment of marker distribution through the dip test and marker spread test. **B** Result of metaclustering of the example dataset, split in 2, 4 or 8 populations. **C** Marker distributions in the formed metaclusters. Green density plots pass the dip test and marker spread test, red distributions denote failed markers due to non-unimodal distribution. **D** For every metaclustering result (denoted with *i*), all marker distributions for the *m* number of metaclusters are taken together and the fraction of passed distributions is taken. **E** Representative diagnostic plot for a larger dataset. The values of the Unimodality set ${U}_{i}$ are plotted on the *y*-axis versus the number of metaclusters assessed on the *x-*axis in red. A sigmoidal curve (blue) is fitted to this data. A plateau is reached where the fraction of unimodal distributions scarcely increases with increasing numbers of metaclusters

The aim of INFLECT is to provide a data-driven evaluation of metaclustering results and calculate the optimal metacluster number where marker distribution quality is balanced with the number of identified clusters. It is written in R and made available here: www.github.com/jnverhoeff/GarciaVallejoLab/INFLECT. The process of INFLECT is illustrated in Fig. 1. In summary, the method takes as input a high-dimensional dataset clustered using the FlowSOM wrapper function, and a set of metacluster targets to be evaluated. The output consist of diagnostic graphs and a determined optimal metacluster number. In the following sections we describe the steps implemented in the R package.

### Iterative metaclustering automation

The process starts with calculating the metaclustering of clusters formed by self-organizing maps of FlowSOM. To ensure capture of the smallest subset of interest, we recommend an initial number of SOM-clusters of at least 200. The subsequent maximum number of metaclusters *k* cannot exceed 90% of the number of SOM-clusters due to resampling within ConsensusClusterPlus. This is done according to either ConsensusClusterPlus[23] or hierarchical clustering based on Minkowski distance and Ward’s linkage. To limit the computation time not every possible metacluster number is evaluated. Using the default settings, INFLECT increases the metacluster targets number by 5 from 50 metaclusters on, and by 10 from 150 metaclusters on. Target numbers are sparser at higher metacluster numbers, due to increased computational load. In our experience the changes between metaclustering results at higher target numbers vary less and thus contribute less information. However, users can specify the amount of metacluster numbers to evaluate using the input vector $set\_i$. This step can be multithreaded to expedite calculations.

### Metaclustering evaluation step

Each *metaclustering result* is evaluated separately, again enabling multithreading. For each metacluster per *metaclustering result*, all relevant marker distributions are evaluated for inter-quartile range and unimodality through the dip test. Results are collected in a matrix of *m* (= the amount of metaclusters) by *n* (= the amount of markers) on a pass/fail basis. A marker distribution fails if the inter-quartile range exceeds a given threshold or has a non-unimodal distribution. Default inter-quartile range threshold is set at a value of 2 after hyperbolic arcsine transformation of raw data (the standard transformation for mass cytometry data). The fraction of distributions that passed this quality control (QC) across all clusters is calculated and given as output *U*. Taken together the outputs form the set ${U}_{i}$, called the Unimodality set.

### Diagnostic plotting

The values within the Unimodality set ${U}_{i}$, are plotted against the number of metaclusters. Subsequently, an L-function is applied to find the inflection point, where dividing the graph in two halves using straight fitted lines results in the least error. This can be done on the Unimodality set itself, or a sigmoidal curve fitted to the data. This implementation of the L-method is based on work by Salvador and Chan [24]. The horizontal coordinate of this inflection point is the optimal metacluster number for this dataset.

## Application to public datasets

We implemented INFLECT on multiple publicly available datasets to assess robustness and applicability. Characteristics of the datasets used are summarized in Table 1. Labels for the events are taken from the datasets directly. In direct comparison of labels and INFLECT metaclusters only labeled data is considered.

**Table 1:** Dataset characteristics

Dataset	Type of samples	Number of cells	Number of labeled cells	Clustering method	Number of labels	Number of markers used in clustering
Levine32 [10]	BMMC^a^	161,443	104,184	Phenograph	14	32
Bagwell [26]	PBMC^b^	101,963	901,559	Manual gating	26	29
KimmeyBM [25]	BMMC	994,897	994,897	SPADE^c^	33	32
KimmeyPBMC [25]	PBMC	795,428	795,428	SPADE	9	12

^a^Bone marrow mononuclear cells

^b^Peripheral blood mononuclear cells

^c^Spanning-tree progression analysis of density-normalized events

INFLECT requires a single input-parameter, $k$, for the amount of clusters generated in the initial SOM-clustering. In the 4 datasets included in this study, the plateau in unimodal marker distributions was consistently reached before $k=100$. For statistical power in the plateau we recommend a value of $k$ of circa 200. In our experiments, to ensure we reached the point of spurious over-clustering, we applied INFLECT to the datasets with $k>350$.

All computations in this work were carried out using a 2X Intel® E5-2660 v3 computing cluster, clocked at 2.60 GHz and 128 GB of RAM. Where possible, processes were run in parallel in 10 threads.

## Validation

KimmeyPBMC used 12 markers for clustering into 9 major phenotypes, whereas the panel by Bagwell uses 29 out of 30 markers for manual gating into 26 phenotypes. This variability revealed the broad applicability of INFLECT, where a broad range of mass cytometry datasets show formation of a stable plateau in metacluster unimodality.

The labeled data allowed for comparison of the inflection point metacluster number to the labeled populations. The number of labels was treated as a measure of dataset heterogeneity. Deviations between INFLECT and the number of labels were further investigated for causes and possible cellular heterogeneity within labels.

## Results

### Unimodal marker distribution of FlowSOM clusters reaches a plateau (for both methods of metaclustering)

Firstly, we investigated how the number of metaclusters related to cluster unimodality and if this could lead to an optimal metacluster number. To assess this we applied INFLECT to 4 publically available datasets. Because FlowSOM requires an input parameter defining the number of resulting clusters, we could iteratively increase the number of generated metaclusters in repeated runs.

**Fig. 2:**
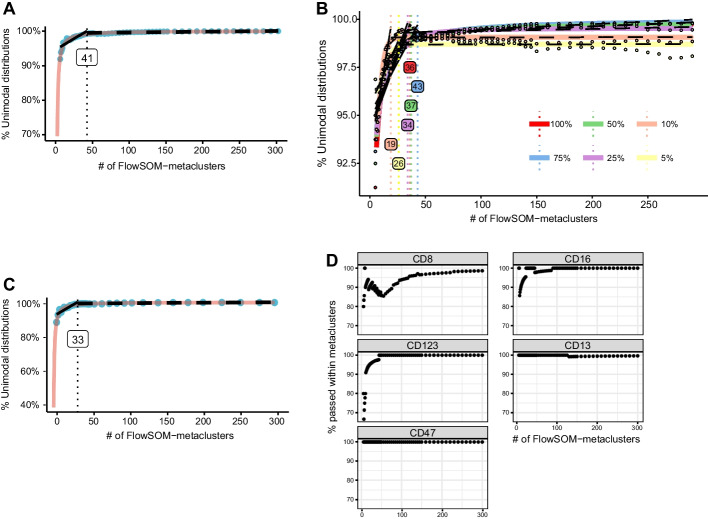
Resulting diagnostic graphs of INFLECT on the Levine32 benchmark dataset. The Unimodality set (${U}_{i}$) is plotted versus the number of metaclusters generated through metaclustering of SOM-clusters. Applying the L-function returns the inflection point where fraction of unimodal distribution plateaus for increasing numbers of metaclusters. **A** Diagnostic graph using hierarchical clustering for metaclustering. The L-function is applied to the fitted curve, resulting in an inflection point of 41 metaclusters. Value for ${U}_{41}$ is determined at 98.17%. **B** Diagnostic graph comparing results for the entire Levine32 dataset using hierarchical clustering to five smaller subsampled datasets, 75%, 50%, 25%, 10% and 5% of events. Unimodality plateaus and inflection points for dataset sizes of 25–100% are consistent. 10% and 5% sizes display a lower plateau and lower inflection point. Of note is that for these smaller datasets, the fraction of unimodal distributions at higher metacluster numbers is less stable. **C** Diagnostic graph using ConsensusClusterPlus metaclustering and L-function on the fitted curve. Due to the longer runtimes of ConsensusClusterPlus fewer ${U}_{i}$ were calculated. Resulting inflection point is 33 metaclusters with ${U}_{33}$ at 98.76%. **D** Marker performance diagnostic plot. For 5 selected markers the fraction of metaclusters which passed the dip test and marker spread test is collected per number of metaclusters. Data is shown in a scatterplot, amount of FlowSOM-metaclusters on the *x*-axis and percentage of metaclusters that passed the unimodality and interquartile range checks. Some (CD8, CD123, CD16) fail the unimodality tests at lower metacluster numbers, while reaching 100% at a higher metacluster numbers. CD13 and CD47 prove very stable at 100%. CD8 displays the highest variability and does not reach 100%, indicating poorer clustering performance for this marker

By evaluating cluster uniformity as a fraction of unimodal markers across clusters, using the dip test for unimodality, we monitored clustering success. In the four labeled datasets we encountered a consistent formation of a plateau. At these plateaus, a (further) increase of metaclusters yielded only a small improvement in cluster unimodality. The Unimodality set ${U}_{i}$ is the combined set of cluster unimodality fractions for every metaclustering *i*. After fitting a sigmoidal curve we implemented the L-function [24] method to calculate the inflection point of the curve. To assess the effects of dataset size and different methods of metaclustering, we applied INFLECT with different combinations of subsamplings of the Levine32 dataset and metaclustering methods as shown in Fig. 2. Different methods of metaclustering yield slightly different sets of ${U}_{i}$ for the same dataset (Hierarchical clustering in Fig. 2A and ConsensusClusterPlus in 2C). The resampling processes in ConsensusClusterPlus [23] are a computationally expensive step, leading to high runtimes for higher values of $i$. Run times are summarized in Table 2.

**Table 2:** Runtime in seconds of metaclustering methods of FlowSOM. k = 375 SOMclusters

Time in seconds	Total ${U}_{i}$		${U}_{325}$	
	Processor time	Elapsed time	Processor time	Elapsed time
Consensus cluster plus, 10 core multithreaded	8853.69	2865.79	2559.75	2582.52
Hierarchical clustering	5.75	5.79	0.05	0.10

For hierarchical clustering $i=(\mathrm{5,6},7\dots 150, 155, 160\dots 200, 210, 220\dots 320)$ were calculated in sequence. ConsensusClusterPlus was calculated for $i = (5, 6, 7\dots 50, 55, 60\dots 100, 120, 140\dots 200, 225, 250, 275, 300)$. Therefore, we applied the much faster hierarchical clustering in all subsequent analysis.

### The metacluster unimodality results ${{\varvec{U}}}_{{\varvec{i}}}$ are dependent on dataset size

To investigate the effect of the size of the data set, we generated smaller datasets by subsampling Levine32 (Fig. 2B). For the full size dataset down to 25% size, the inflection points and unimodality score plots are very similar, ranging from 36 metaclusters for the full dataset to 34 for the 25% sample. At 10% size, which is 60,000 cells for this dataset, the inflection point shifts to 19 metaclusters, and to 26 metaclusters for 5% size. Of note is that the cluster unimodality fraction of the plateau for higher metacluster numbers seems to drop for the smaller dataset sizes.

In Fig. 2D we display the marker performance in the dataset across all the metacluster numbers. Several markers pass the unimodality and marker spread checks for all metacluster numbers, such as CD13 or CD47. Other markers show a greater spread, having multi-modal distributions for lower metacluster numbers but rising to 100% for very high metacluster numbers (CD123 and CD16) or never reaching 100% (CD8). This graph can inform researchers on the clustering power of different markers in the dataset. In Additional file 1: Fig. S1, the performance of the remaining clustering markers is shown.

### The L-function provides a data-driven Inflect point for unimodality scores and fitted curves

**Fig. 3:**
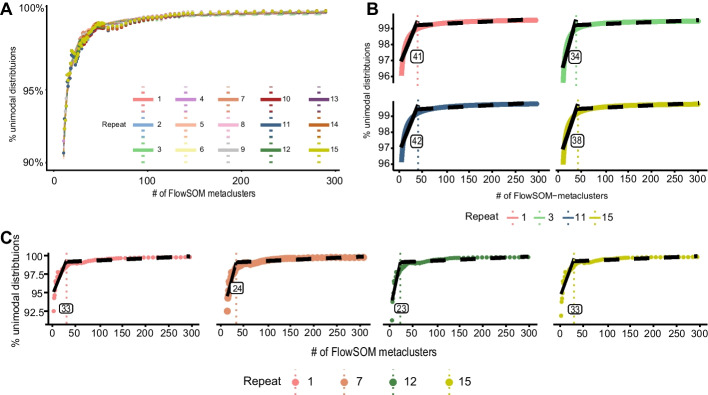
Diagnostic plots of INFLECT generated on 15 independent 90% subsamples of the Levine32 benchmark dataset. **A** Overlaid INFLECT diagnostic plots for each 90% subsample. Unimodality set ${U}_{i}$ points are connected with colored lines. **B** Four selected INFLECT diagnostic plots for 90% subsamples with the greatest range. Shown are the fitted curves and the L-function inflection points generated from the curves. Inflection point indicated with vertical line. **C** Four selected INFLECT diagnostic plots for 90% subsamples with the greatest range. Here, the L-function is applied directly to the data (the Unimodality set ${U}_{i}$), without fitting a sigmoidal curve. All determined inflection points are indicated with a dashed vertical line

To investigate the robustness of INFLECT cluster unimodality and the implementation of the L-function, we subsampled 90% of the Levine32 dataset 15 times. INFLECT was applied on the 15 replicates. Resulting unimodality sets (Fig. 3A) exhibit little variation. In four out of the 85 metacluster numbers evaluated, all 15 replicates yielded the same cluster accuracy. Greatest variability between replicates was 0.69%, which occurred at *m* = 9 metaclusters. Applying the L-function on fitted curves (Fig. 3B) vary between 36 and 42, with a mean of 38.90 and a SEM of 0.62. When applying the L-function directly on the Unimodality set ${U}_{i}$ (Fig. 3C) data points, the position of the inflection point is more vulnerable to noise, with a lower mean of 28 but a higher SEM of 0.90. Repeats shown in Fig. 3 represent the greatest variability between the in total 15 replicates. Remaining repeats are shown in Additional file 1: Fig. S2. In addition to lower variability, fitting a sigmoidal curve to the data lowers the amount of metaclusters that need to be evaluated, further speeding up calculations. In Additional file 1: Fig. S3 we show that even with lower amount of data points in the Unimodality set, the fitted curve provides a stable inflection point. Hence, applying the L-function to fitted curves gives more robust results.

### INFLECT captures underlying heterogeneity in labeled clusters of Levine32

**Fig. 4:**
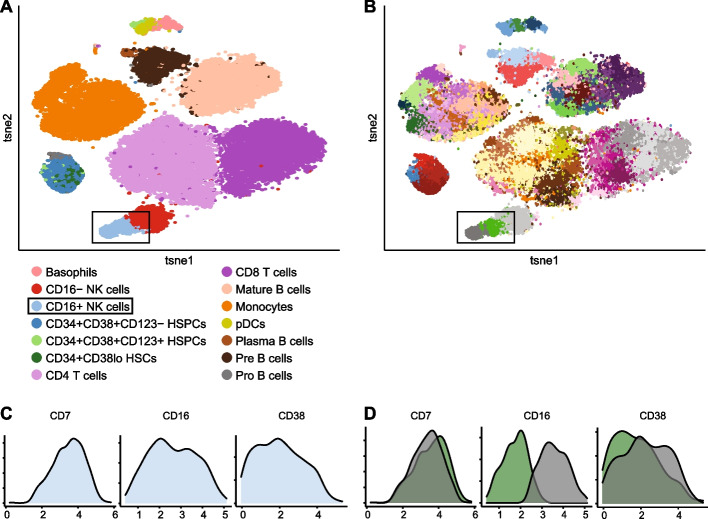
INFLECT identifies additional heterogeneity present within labeled data. **A-B** tSNE embedding of 40,000 events from Levine32, in **A** colored for the 14 phenotypes as identified by Phenograph in the original paper. In **B**, 61 metaclusters identified through INFLECT. **C** Density plots of selected markers for CD16 + NK cells. *X*-axis denote Arcsinh(x/5) transformed expression values. **D** Density plots of selected markers highlighting heterogeneity within CD16 + NK cells, which was split into metaclusters 60 and 61. *X*-axis denote Arcsinh(x/5) transformed expression values. Metacluster 60 has a CD16-dim phenotype, whereas metacluster 61 has high CD16 expression

For visualization purposes we sampled 40,000 events from Levine32 and performed a tSNE embedding (Fig. 4A). As can be expected, INFLECT splits several of the more abundant populations into smaller metaclusters, while matching smaller distinct cell types such as CD16- natural killer (NK) cells and Plasma B cells to one metacluster each. In this case INFLECT was run only on labeled data, which was 65% of total events. The optimal metacluster number for this data was higher, at 61 metaclusters, shown in Fig. 4B. In Additional file 1: Fig. S4, we compare INFLECT to two common clustering evaluation tools [27], and the default method of metaclustering in FlowSOM. The 2 metaclusters most closely matching CD16 + NK cells were investigated (metacluster 60 and 61) through visual inspection of marker histograms (Fig. 4C and D). 3 markers were selected for illustrating the greatest variability between the metaclusters. CD16 + NK cells show no multi-modality as a total population, passing the dip test for each marker distribution, however in Fig. 4D it is shown that metaclusters 60 and 61 have distinct levels of CD16 expression. This seems to correspond well to the tSNE mapping in 4A, where the light blue CD16 + NK cell population consists of two connected populations. Full concordance between labeled data and INFLECT metaclusters is summarized in Additional file 2.

### The inflection point is consistent across mass cytometry datasets

**Fig. 5:**
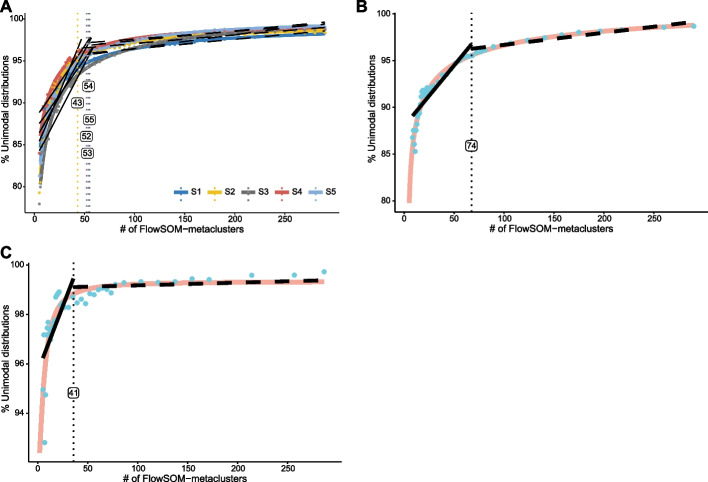
INFLECT Unimodality plateaus are reached across mass cytometry data sets. **A** INFLECT Diagnostic curve for five replicate PBMC samples from Bagwell et al. Samples were clustered and evaluated separately and inflection points were determined. **B** KimmeyBM INFLECT diagnostic curve. Inflection point (= 74) is calculated on the fitted sigmoidal curve. **C** KimmeyPBMC INFLECT diagnostic curve. Inflection point (= 41) is calculated on the fitted sigmoidal curve

Wider applicability of INFLECT was investigated by applying the method to 3 other datasets (Fig. 5). In the Bagwell PBMC dataset (Fig. 5A) all 5 samples (which are technical replicates acquired at different sites) showed a similar pattern of cluster unimodality. The fraction of unimodal distributions rose rapidly to 95% for all 5 samples. The fraction of unimodal distributions increased at a lower rate for increasing numbers of clusters, reaching 99.02% unimodality for 300 FlowSOM metaclusters. Applying the L-function output a median of 53 metaclusters as the optimal number of metaclusters for this dataset with a fraction of unimodal distributions ${U}_{53}= 95.61\%$. Diagnostic curve for KimmeyBM (Fig. 5B) showed a similar pattern, ${U}_{5\dots 73}$ rising to 95.60% unimodality, then yielded diminishing returns up to 99.70% fraction of unimodal distributions. Inflection point, resulting from the L-function was at 74 metaclusters.. The KimmeyPBMC dataset had fewer markers used for phenotyping than the bone marrow dataset. This resulted in a lower optimal cluster number, determined at 41 metaclusters with a fraction of unimodal distributions of 98.89%. For higher numbers of metaclusters, the diagnostic curve had a nearly level unimodality plateau starting at inflection point = 42. Unimodality increases up to 98.37% at ${U}_{42}$, ultimately reaching 99.70% at ${U}_{350}$.

Similar to the Levine32 dataset, the Bagwell and Kimmey datasets are labeled by the original authors. Comparisons between the original labels and INFLECT-informed FlowSOM metaclusters were made using tSNE embeddings, marker histograms, and concordance between original labels and INFLECT metaclusters are shown via matching matrixes (Additional file 1: Figs. S5–S7, Additional file 3–5).

For the Bagwell dataset labels were determined by a published sequential gating strategy, though overlapping gates lead to events receiving multiple labels. INFLECT was able to identify a small population of CD16^low^ and CD56^high^ NK cells within the Early NK cell label (Additional file 1: Fig. S5C-D). Concordance between event labels and INFLECT metaclusters is summarized in Additional file 3.

Both datasets in the paper by Kimmey et al. were labeled by manually annotating a large number of SPADE clusters, 175 clusters for the bone marrow dataset and 150 clusters for the PBMC dataset. Concordance between event labels and INFLECT metaclusters are displayed in Additional file 4 and 5, and visualized in Figs. S6 and S7 (Additional file 1). In both datasets INFLECT was again able to identify phenotypically distinct populations within labels.

Across all datasets considered in this work, the fraction of unimodal distributions reaches a plateau of diminishing returns. The start of this plateau can be determined by the L-function, providing an optimal metacluster number for the particular dataset.

## Discussion

In this work we outline the use of overall cluster unimodality as a quality control measurement for FlowSOM clustering results. The goal of clustering is to determine and enumerate unique cellular subsets, or in other words grouping cells with unimodal distributions in all markers. By using cluster unimodality this clustering process can be condensed to a single score for each set of FlowSOM results, collected in the Unimodality set ${U}_{i}$. The chosen datasets contain labels for known cell phenotypes through different analysis methods, each with its own advantages and assumptions. The datasets also vary in dimensionality and depth of phenotyping. This underscores the flexibility of INFLECT. The fraction of unimodal distributions for a dataset reaches a plateau, at an inflection point that is dependent on dataset size, number of markers, and expression patterns of markers. While use of marker unimodality has been described before in high-dimensional analysis [11], many rely on Gaussian mixture modeling or expectation maximization [28–31]. These processes are computationally expensive, making them impractical for use in a quality control setting where we would want to evaluate iterative clustering runs. Moreover, unimodality testing through the dip test has been described as more stable and less prone to errors in literature [19].

This work is inspired by quality control functions that are part of SPADEVizR [18] and work on the L-function [24]. The hyperparameters used in INFLECT, the threshold for interquartile range (set at $arcsinh(X/5)= 2$) and the dip test ($\alpha = 0.05$) are the same as in SPADEVizR. When combined into INFLECT we show that they provide a data-driven metaclustering endpoint for FlowSOM. Though not investigated in this study, the application of INFLECT should work for any clustering method where the user can determine the number of resulting clusters. This can be either in cases where the number of clusters is used as input, such as SCAFFoLD [32] or where the smallest acceptable cluster is determined as in Citrus [12]. Additionally, there are methods where input from the user more indirectly affects cluster composition and total number of clusters, such as bandwidth selection in density-based clustering (ACCENSE [33] or Gaussian mean shift-based clustering [34]). INFLECT can be utilized to iteratively evaluate these input parameters aside from visual inspection of tSNE embeddings. These approaches come with the caveat that they likely require take a longer time to compute. The design of FlowSOM allows for low runtimes also for larger datasets [21], making it very suitable for testing many iterations to determine the best clustering. Applying INFLECT to clustering algorithms other than FlowSOM was outside of the scope of this work and would have to be validated before implementation.

While INFLECT was developed for use in mass cytometry, the principle behind cluster unimodality would still hold true for high-dimensional cytometry techniques like spectral cytometry [35] or high-parameter flow cytometry. However, extra care should be taken in pre-processing of the data regarding distributions around zero (and lower). Since mass cytometry has no negative values, the unimodality test is performed on positive distributions only. Unexpected minor cross-talk between markers in conventional flow cytometry could result in multi-modal negative peaks and will likely lead INFLECT to recommend an optimal cluster number that is too high [36, 37]. The distributions of gene expressions in scRNA-seq data are not comparable to protein expression in mass cytometry. Therefore we do not expect INFLECT to provide a benefit when assessing the clustering results of single cell RNA sequencing (scRNA-seq) data.

When comparing INFLECT clustering with the cluster definitions as originally published for the dataset, the inflection point was consistently higher than the number of phenotypes determined by the authors. By visually inspecting histograms for representative INFLECT-metaclusters, we show that the clusters have distinct marker expression patterns. While it is unlikely that a wholly novel cell type is discovered, by splitting cell types into distinct sub-clusters INFLECT enumerates possible differentiation states or activation states. INFLECT thereby allows for differential abundance testing on these sub-clusters. However, it is still up to the researcher to determine if this cluster of events represents a biologically meaningful subset. For example the CD56^bright^ population of NK cells identified within the BMMC dataset of Levine et al., while small, is an important subset with a distinct function in the immune system [38]. The rationale for finding the data-driven endpoint of clustering is to limit the number of clusters, in turn to limit the number of statistical tests that will be performed and reduce occurrence of type 1 errors. Therefore, manually merging clusters that were found in an unsupervised manner is a valid strategy. The diagnostic curves provided by INFLECT can aid in this process, showing at what number of metaclusters cluster unimodality will drop dramatically.

## Conclusion

INFLECT addresses a knowledge gap in high-dimensional cytometry analysis, namely assessing clustering results. This is done through monitoring marker distributions for interquartile range and unimodality across a range of metacluster numbers. The fraction of unimodal distributions within metaclusters, collected in the Unimodality set ${U}_{i}$, plotted versus the number of clusters consistently reaches a plateau, providing a data-driven endpoint for metacluster number. The inflection point is the optimal trade-off between cellular heterogeneity and statistical power, applied in this work for FlowSOM clustering on mass cytometry datasets.

### Availability and requirements

The computational method proposed in this work is collected in the R package “INFLECT” and made available on github at www.github.com/jnverhoeff/GarciaVallejoLab. Project name: INFLECT. Project home page: https://www.github.com/jnverhoeff/GarciaVallejoLab. Operating system(s): Platform independent. Programming language: R. Other requirements: R4.1.2. License: GNU GPLv3. Any restrictions to use by non-academics: license needed for commercial use.

## Supplementary Information

**Additional file 1: Supplementary Fig. S1:** Marker performance for Levine32 INFLECT. Marker performance diagnostic plots for remaining markers of Levine32. On the *x*-axis the amount of FlowSOM-metaclusters, and on the *y*-axis the amount of metaclusters that pass the unimodality and interquartile range checks as a percentage of total metaclusters for the listed marker; **Supplementary Fig. S2:** All 15 repeats of 90% subsamples of Levine32. **A** L-function applied to the sigmoidal curve fitted to set${U}_{i}$. **B** L-function applied to the set ${U}_{i}$ itself. Resulting inflection points for both panels denoted with vertical dotted line; **Supplementary Fig. S3:** With fewer datapoints in Unimodality set ${U}_{i}$ INFLECT still produces a stable fitted sigmoidal curve. INFLECT diagnostic plots with different sizes of set ${U}_{i}$, from 81 calculations down to 9 datapoints. Resulting inflection point is stable around 40 metaclusters. The values of the Unimodality set ${U}_{i}$ are plotted on the *y*-axis versus the number of metaclusters assessed on the *x-*axis; **Supplementary Fig. S4:** Traditional (meta)clustering evaluations perform poorly on Levine32 dataset. **A** Davies Bouldin (DB) Index on the *y*-axis versus the amount of FlowSOM-metaclusters. A lower score indicates better clustering. In this case the DB index does not form a plateau, making interpretation difficult. **B** Calinski-Harabasz (CH) Index on the *y*-axis versus the amount of FlowSOM-metaclusters. A higher score indicates better clustering. CH index drops with increasing FlowSOM-metaclusters, making CH index less suitable for evaluation. **C** Diagnostic plot of ConsensusClusterPlus on Levine32 dataset. Relative change in area under cumulative distribution function (CDF) curve compared to $k-1$ clusters. FlowSOM implementation of ConsensusClusterPlus indicates 14 metaclusters as optimal$k$. **D** tSNE embedding of Levine32, colored for the 14 ConsensusClusterPlus metaclusters. Multiple islands (CD4 T cells, CD8 T cells and monocytes) are grouped into 1 large metacluster; **Supplementary Fig. S5:** INFLECT reveals heterogeneity within labeled phenotypes of the replicate PBMC dataset from Bagwell et al. **A** tSNE embedding of 10,000 events from Bagwell dataset. Colored for the 26 manually gated phenotypes, plus light-pink for unlabeled cells and yellow for events with 2 or more labels. **B** Histograms of selected markers highlighting heterogeneity within the Early NK cells. *X*-axis denote Arcsinh(x/5) transformed expression values. **C** Same tSNE embedding of 10,000 events as in **A**, here colored for the 52 INFLECT metaclusters. **D** Histograms of selected markers highlighting the difference between metacluster 24 and 29, which correspond to the 2 different cell populations within the Early NK label. *X*-axis denote Arcsinh(x/5) transformed expression values. Metacluster 29 is a smaller CD56^bright^ CD16^dim^ population, whereas metacluster 24 has high levels of CD16 and intermediate CD56 expression; **Supplementary Fig. S6:** INFLECT applied to bone marrow dataset from Kimmey et al. reveals high degree of heterogeneity. **A** tSNE embedding of 10,000 events from KimmeyBM dataset. Colors denote the labels identified by the authors. Immature B cells are highlighted in rectangle. **B** Histograms of selected markers highlighting heterogeneity within Immature B cells. *X*-axis denote Arcsinh(x/5) transformed expression values. **C** Same tSNE embedding as in **A**, now colored for the 74 INFLECT metaclusters. Highlighted in the rectangle are the 4 different metaclusters of the Immature B cells, which were separated into metaclusters 38, 41, 49, and 71. **D** Histograms of selected markers highlighting heterogeneity between the 4 metaclusters corresponding to the Immature B cell label. *X*-axis denote Arcsinh(x/5) transformed expression values. Metacluster 71 has a CD38^+^ phenotype, metacluster 41 has a IgM^dim^ phenotype and metaclusters 38 and 49 are differentiated in expression levels of CD24 and to a lesser extent CD45RA; **Supplementary Fig. S7:** INFLECT algorithm applied to PBMC dataset from Kimmey et al. captures underlying heterogeneity of labeled populations.** A** tSNE embedding of 10,000 events from KimmeyPBMC dataset. Colors denote the labels identified by the authors. Rectangle highlights the cDCs label. **B** Histograms of selected markers highlighting heterogeneity within the cDCs label. *X*-axis denote Arcsinh(x/5) transformed expression values. **C** Same tSNE embedding as in **A**, here colored for the 41 INFLECT metaclusters. In the rectangle the metaclusters 7 and 15 are highlighted, which correspond to the cDCs label. **D** Histograms of selected markers highlighting heterogeneity between metaclusters 7 and 15. Main difference between metaclusters 7 and 15 is the level of CD11c expression.

**Additional file 2:** Matching matrix of Levine32 dataset.

**Additional file 3:** Matching matrix of Bagwell dataset.

**Additional file 4:** Matching matrix of Kimmey-BM dataset.

**Additional file 5:** Matching matrix of Kimmey-PBMC dataset.

## Data Availability

The datasets supporting the conclusions of this article are available in the Flowrepository, Levine32 under FR-FCM-ZZPH at http://flowrepository.org/id/FR-FCM-ZZPH, both Kimmey et al*.* datasets under FR-FCM-ZYR5 at http://flowrepository.org/id/FR-FCM-ZYR5. The Bagwell et al*.* dataset is available on Cytobank, under Bagwell et al. at http://premium.cytobank.org/cytobank/experiments/221569.

## Abbreviations

QC: Quality control
BMMC: Bone marrow mononuclear cells
PBMC: Peripheral blood mononuclear cell
NK: Natural killer
SPADE: Spanning-tree progression analysis of density-normalized events
scRNA-seq: Cells, single cell RNA sequencing
tSNE: T-distributed stochastic neighbor embedding
UMAP: Uniform manifold approximation projection
SOM: Self-organizing maps
