# Supplementary material for: INFLECT: an R-package for cytometry cluster evaluation using marker modality
Source: BMC Bioinformatics. 2022 Nov 16;23:487. doi: 10.1186/s12859-022-05018-w (PMC9670405; doi:10.1186/s12859-022-05018-w)
Supplement: Supplementary file 1 — Additional file 1: Supplementary Fig. S1: Marker performance for Levine32 INFLECT. Marker performance diagnostic plots for remaining markers of Levine32. On the x-axis the amount of FlowSOM-metaclusters, and on the y-axis the amount of metaclusters that pass the unimodality and interquartile range checks as a percentage of total metaclusters for the listed marker; Supplementary Fig. S2: All 15 repeats of 90% subsamples of Levine32. A L-function applied to the sigmoidal curve fitted to set${U}_{i}$Ui. B L-function applied to the set ${U}_{i}$Ui itself. Resulting inflection points for both panels denoted with vertical dotted line; Supplementary Fig. S3: With fewer datapoints in Unimodality set ${U}_{i}$Ui INFLECT still produces a stable fitted sigmoidal curve. INFLECT diagnostic plots with different sizes of set ${U}_{i}$Ui, from 81 calculations down to 9 datapoints. Resulting inflection point is stable around 40 metaclusters. The values of the Unimodality set [file 12859_2022_5018_MOESM1_ESM.pdf]

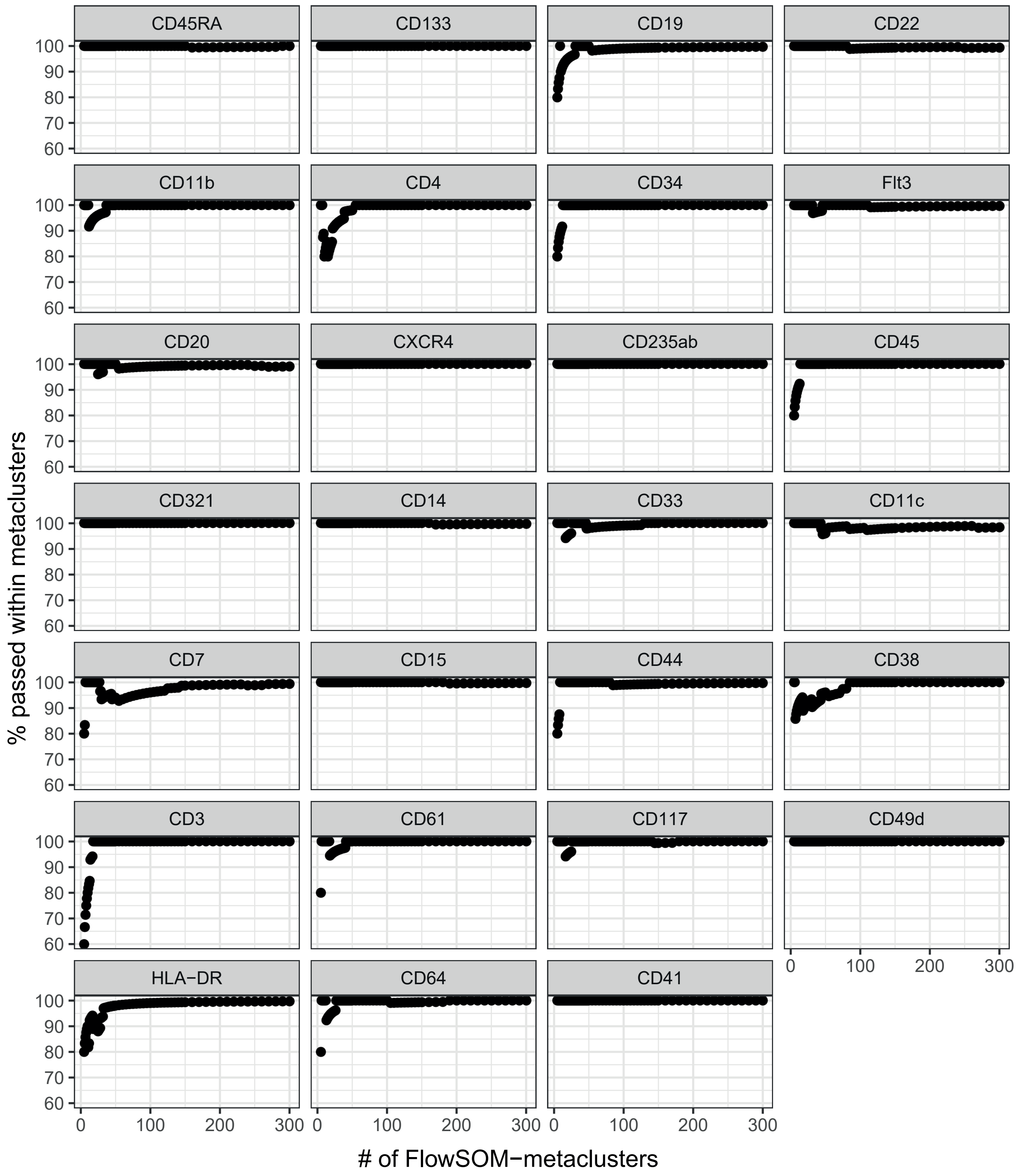

Supplementary figure 1 - Marker performance for Levine32 INFLECT.
Marker performance diagnostic plots for remaining markers of Levine32. On the x-axis the amount of FlowSOM-metaclusters, and on the y-axis the amount of metaclusters that pass the unimodality and interquartile range checks as a percentage of total metaclusters for the listed marker.

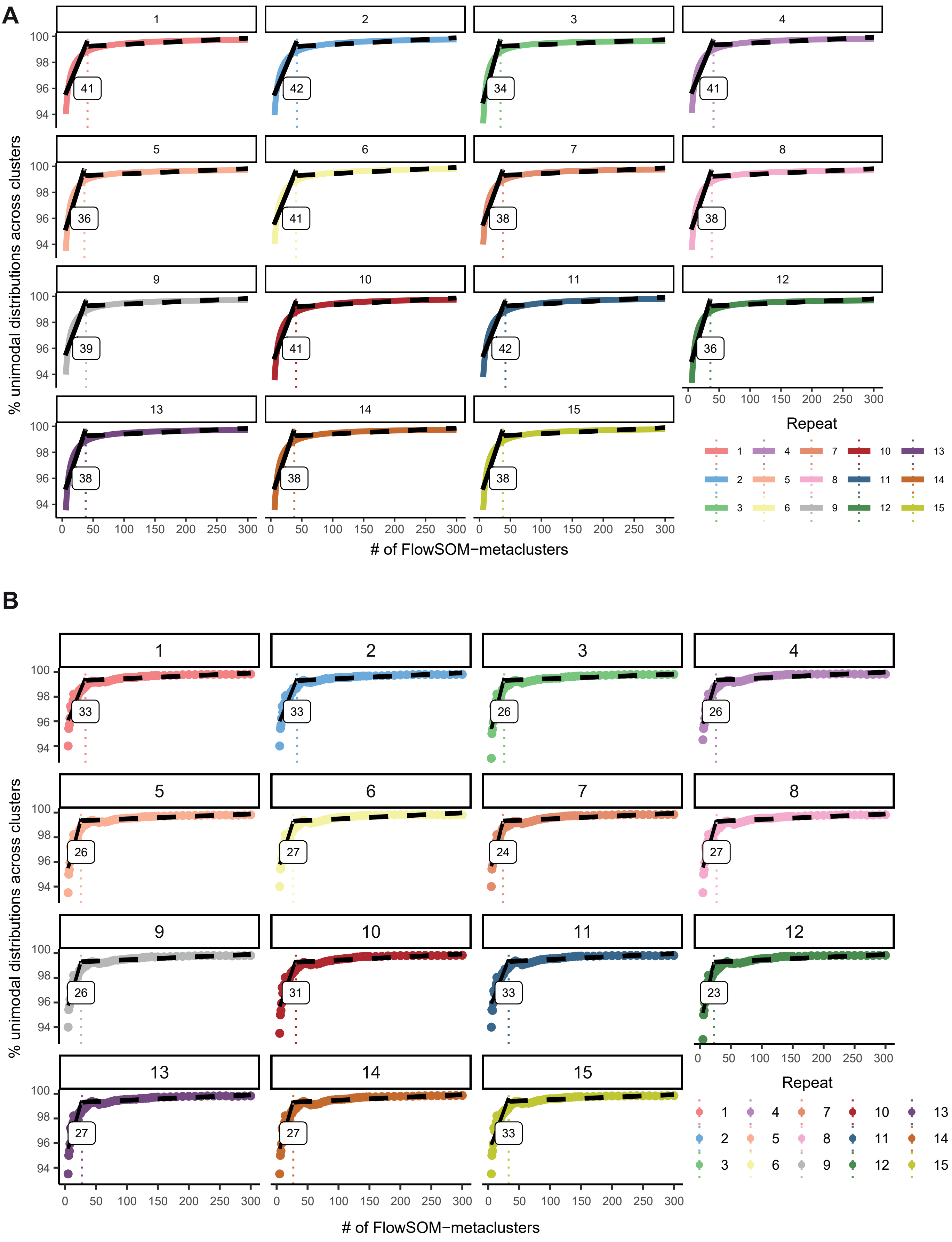

Supplementary figure 2 - All 15 repeats of 90% subsamples of Levine32.
A L-function applied to the sigmoidal curve fitted to set U_i. **B** L-function applied to the set U_i itself. Resulting inflection points for both panels denoted with vertical dotted line.

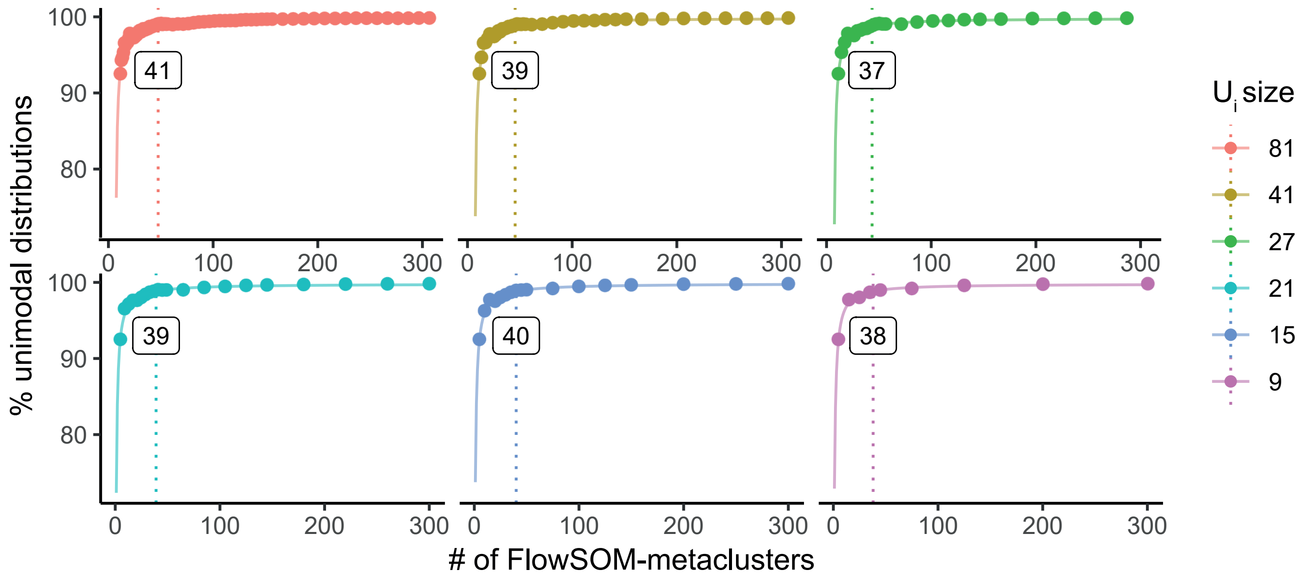

Supplementary figure 3 - With fewer datapoints in Unimodality set U_i INFLECT still produces a stable fitted sigmoidal curve.
INFLECT diagnostic plots with different sizes of set U_i , from 81 calculations down to 9 datapoints. Resulting inflection point is stable around 40 metaclusters. The values of the Unimodality set U_i are plotted on the y-axis versus the number of metaclusters assessed on the x-axis.

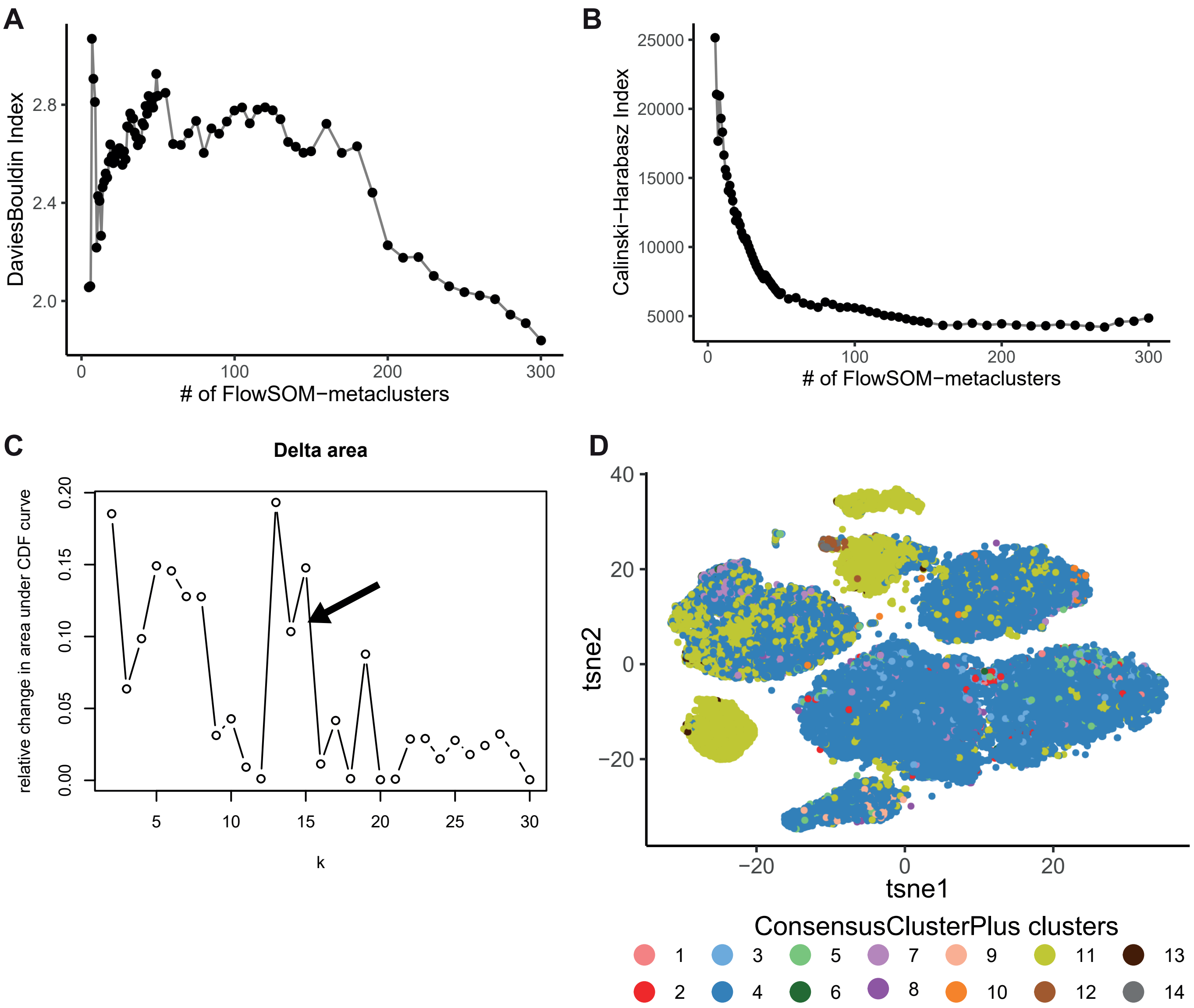

Supplementary figure 4 - Traditional (meta)clustering evaluations perform poorly on Levine32 dataset.

A Davies Bouldin (DB) Index on the y-axis versus the amount of FlowSOM-metaclusters. A lower score indicates better clustering. In this case the DB index does not form a plateau, making interpretation difficult. **B** Calinski-Harabasz (CH) Index on the y-axis versus the amount of FlowSOM-metaclusters. A higher score indicates better clustering. CH index drops with increasing FlowSOM-metaclusters, making CH index less suitable for evaluation. **C** Diagnostic plot of CCP on Levine32 dataset. Relative change in area under cumulative distribution function (CDF) curve compared to k-1 clusters. FlowSOM implementation of CCP indicates 14 metaclusters as optimal k. **D** tSNE embedding of Levine32, colored for the 14 CCP metaclusters. Multiple islands (CD4 T cells, CD8 T cells and monocytes) are grouped into 1 large metacluster.

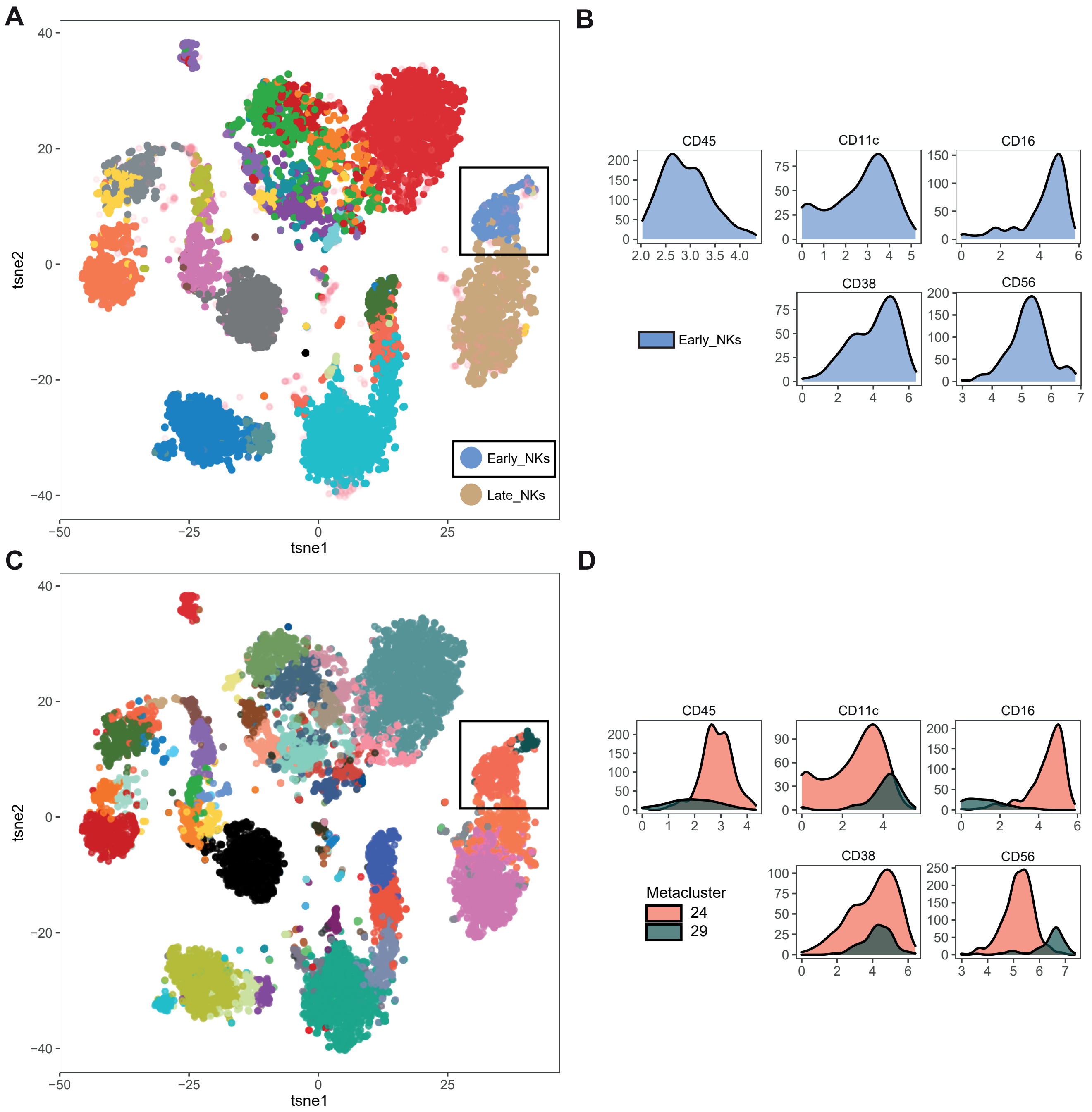

Supplementary figure 5 - INFLECT reveals heterogeneity within labeled phenotypes of the replicate PBMC dataset from Bagwell et al.

A tSNE embedding of 10,000 events from Bagwell dataset. Colored for the 26 manually gated phenotypes, plus light-pink for unlabeled cells and yellow for events with 2 or more labels.
B Histograms of selected markers highlighting heterogeneity within the Early NK cells. X-axis denote Arcsinh(x/5) transformed expression values. **C** Same tSNE embedding of 10,000 events as in A, here colored for the 52 INFLECT metaclusters. **D** Histograms of selected markers highlighting the difference between metacluster 24 and 29, which correspond to the 2 different cell populations within the Early NK label. X-axis denote Arcsinh(x/5) transformed expression values. Metacluster 29 is a smaller CD56bright CD16dim population, whereas metacluster 24 has high levels of CD16 and intermediate CD56 expression.

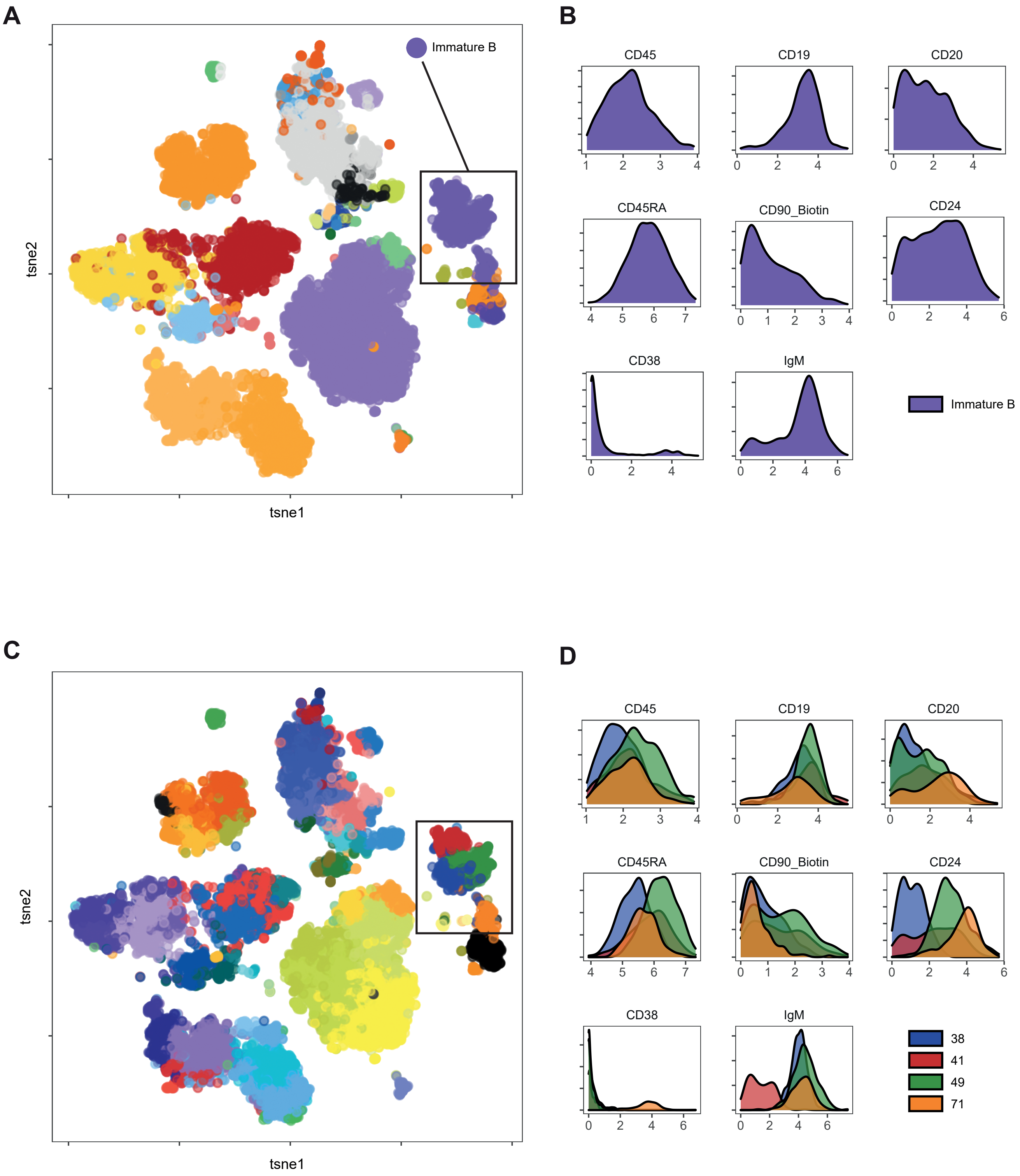

Supplementary figure 6 - INFLECT applied to bone marrow dataset from Kimmey et al. reveals high degree of heterogeneity.

A tSNE embedding of 10,000 events from KimmeyBM dataset. Colors denote the labels identified by the authors. Immature B cells are highlighted in rectangle. **B** Histograms of selected markers highlighting heterogeneity within Immature B cells. X-axis denote Arcsinh(x/5) transformed expression values. **C** Same tSNE embedding as in A, now colored for the 74 INFLECT metaclusters. Highlighted in the rectangle are the 4 different metaclusters of the Immature B cells, which were separated into metaclusters 38,41,49, and 71. **D** Histograms of selected markers highlighting heterogeneity between the 4 metaclusters corresponding to the Immature B cell label. X-axis denote Arcsinh(x/5) transformed expression values. Metacluster 71 has a CD38+ phenotype, metacluster 41 has a IgMdim phenotype and metaclusters 38 and 49 are differentiated in expression levels of CD24 and to a lesser extent CD45RA.

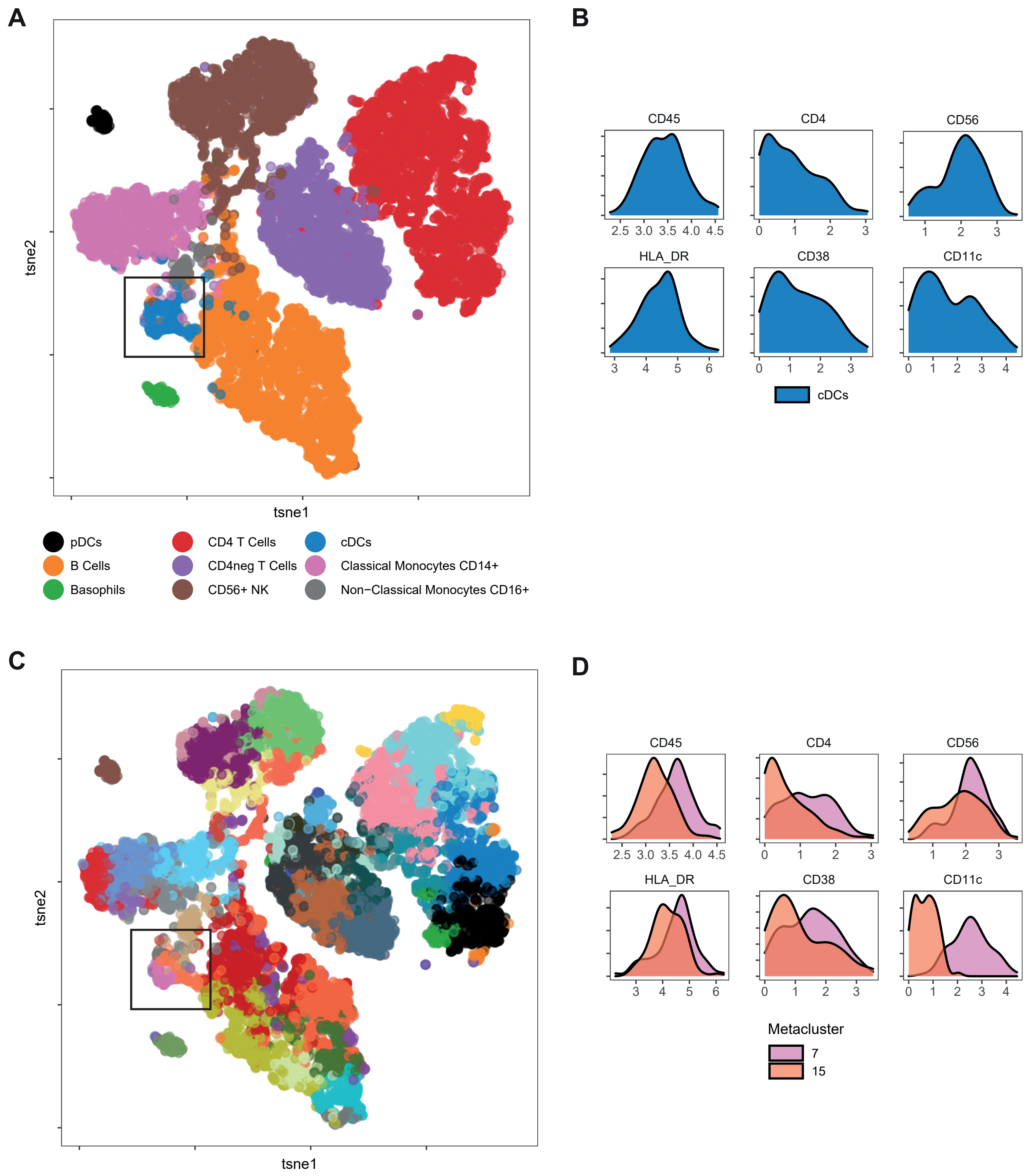

Supplementary figure 7 - INFLECT algorithm applied to PBMC dataset from Kimmey et al captures underlying heterogeneity of labeled populations.

A tSNE embedding of 10,000 events from KimmeyPBMC dataset. Colors denote the labels identified by the authors. Rectangle highlights the cDCs label. **B** Histograms of selected markers highlighting heterogeneity within the cDCs label. X-axis denote Arcsinh(x/5) transformed expression values. **C** Same tSNE embedding as in A, here colored for the 41 INFLECT metaclusters. In the rectangle the metaclusters 7 and 15 are highlighted, which correspond to the cDCs label. **D** Histograms of selected markers highlighting heterogeneity between metaclusters 7 and 15. Main difference between metaclusters 7 and 15 is the level of CD11c expression.
